# A tomato NAC transcription factor, SlNAP1, directly regulates gibberellin-dependent fruit ripening

**DOI:** 10.1186/s11658-024-00577-7

**Published:** 2024-04-23

**Authors:** Changxia Li, Xuemei Hou, Zongxi Zhao, Huwei Liu, Panpan Huang, Meimei Shi, Xuetong Wu, Rong Gao, Zhiya Liu, Lijuan Wei, Yihua Li, Weibiao Liao

**Affiliations:** 1 College of Horticulture, Gansu Agricultural University, 1 Yinmen Village, Anning District, Lanzhou, 730070 China; 2 College of Agriculture, Guangxi University, 100 East University Road, Xixiangtang District, Nanning, 530004 China; 3 Spice Crops Research Institute, College of Horticulture and Gardening, Yangtze University, Jingzhou, 434025 China

**Keywords:** NAC, Gibberellin, Tomato, Transcription factor, Fruit ripening, Protein–protein interaction

## Abstract

In tomato (*Solanum lycopersicum*), the ripening of fruit is regulated by the selective expression of ripening-related genes, and this procedure is controlled by transcription factors (TFs). In the various plant-specific TF families, the no apical meristem (NAM), *Arabidopsis thaliana* activating factor 1/2 (ATAF1/2), and cup-shaped cotyledon 2 (CUC2; NAC) TF family stands out and plays a significant function in plant physiological activities, such as fruit ripening (FR). Despite the numerous genes of NAC found in the tomato genome, limited information is available on the effects of NAC members on FR, and there is also a lack of studies on their target genes. In this research, we focus on SlNAP1, which is a NAC TF that positively influences the FR of tomato. By employing CRISPR/Cas9 technology, compared with the wild type (WT), we generated *slnap1* mutants and observed a delay in the ethylene production and color change of fruits. We employed the yeast one-hybrid (Y1H) and dual-luciferase reporter (DLR) assays to confirm that SlNAP1 directly binds to the promoters of two crucial genes involved in gibberellin (GA) degradation, namely *SlGA2ox1* and *SlGA2ox5*, thus activating their expression. Furthermore, through a yeast two-hybrid (Y2H), bimolecular fluorescence complementation (BIFC) and luciferase (LUC) assays, we established an interaction between SlNAP1 and SlGID1. Hence, our findings suggest that SlNAP1 regulates FR positively by activating the GA degradation genes directly. Additionally, the interaction between SlNAP1 and SlGID1 may play a role in SlNAP1-induced FR. Overall, our study provides important insights into the molecular mechanisms through which NAC TFs regulate tomato FR via the GA pathway.

**Supplementary Information:**

The online version contains supplementary material available at 10.1186/s11658-024-00577-7.

## Introduction

The progression of tomato (*Solanum lycopersicum*) fruit ripening (FR) is controlled by different transcription factors (TFs), specifically NAC [no apical meristem (NAM), *Arabidopsis thaliana* activating factor 1/2 (ATAF1/ATAF2), and cup-shaped cotyledon 2 (CUC2)], MADS (minichromosome maintenance 1 (MCMI), AGAMOUS (AG), defensin alpha (DEFA), and serum response factor (SRF)], Cys2-His2 (C2H2) zinc finger proteins (ZFPs), and basic helix–loop–helix (bHLH) [1–4]. These TFs oversee the maturation of tomato FR by binding to the promoters of specific genes linked to ethylene (ETH) biosynthesis (*SlACS2* and *SlACS4*), abscisic acid (ABA) synthesis (*SlNECD*), color pigmentation regulated by *SlSGR1*, and the metabolism of the cell wall (*SlPG2a*, *SlPL*, *SlCEL2*, and *SlEXP1*) [5]. Although a variety of TFs are involved in tomato ripening, the detailed function of the transcriptional regulatory network controlling tomato FR has not been fully understood.

The NAC TFs, exclusive to plants, is a prominent gathering of TFs. In the genome of the tomato, a total of 101 NACs have been identified [6]. The regulation of tomato FR is specifically linked to NAC TFs, such as NOR, NOR-like, SlNAC1, SlNAC4, SlNAC9, and SlNAM1 [1, 7–11]. Studies on the involvement of NAC TFs in the process of tomato FR originated from the identification of the spontaneous nonripening (*nor*) mutant [12, 13]. In climacteric FR, the fruit fails to produce significant levels of ETH hormone, resulting in a firm texture and yellow coloration of the fruit [12, 13]. Using map-based cloning techniques, researchers have identified that the *nor* mutant originates from the deletion of two A bases within the NAC-NOR coding sequence (CDS). This deletion consequently results in the premature termination of protein translation, causing the production of a truncated protein [12]. Recent studies have indicated that CRISPR/Cas9-mediated manipulation of the NAC-NOR gene in tomato fruits results in the inability to replicate the phenotype observed in the *nor* mutant, implying that the *nor* mutant may represent a gain-of-function mutation [8, 14]. Further investigations have revealed the presence of a truncated variant of the *nor* mutant protein (NAC-NOR186) within the nucleus, which exhibits binding capabilities to ripening-related gene promoters but lacks the ability to activate them [15]. In addition to the well-known NAC-NOR gene, other NAC TF family members, such as SlNAC1, SlNAC4, SlNAC9, SlNAM1, and SlNOR-like1, have also been associated with the ripening process [8, 9, 11, 16]. According to Manning et al. [17], inhibition of the *SINAC4* gene leads to several outcomes, including reduced ETH synthesis, delayed fruit development, and decreased carotenoid (Car) accumulation. Moreover, SlNAM1, as an NAC TF, plays a crucial role in initiating tomato FR by regulating ETH production [11]. NAC plays a significant role in the physiological and biochemical changes that occur during FR, specifically in the synthesis of ETH. In fact, NAC works in conjunction with other phytohormones to regulate FR. Melatonin (MT), salicylic acid (SA), gibberellin (GA), ETH, ABA, brassinolide (BR), indole-3-acetic acid (IAA), jasmonic acid (JA), and GA are all phytohormones known to influence FR [18]. ETH and ABA are the main hormones involved in the FR of both climacteric and non-climacteric fruits. It has been discovered that NAC TFs play a crucial role in integrating phytohormone signaling and FR in various plant species [19, 20]. For instance, NAC TFs positively regulate the ripening of strawberry fruit by upregulating genes associated with ABA biosynthesis [19]. Similarly, in citrus, CrNCED5 expression is inhibited by the CrNAC036 TF in collaboration with CrMYB68, which suppresses ABA biosynthesis during citrus ripening [20]. The primary focus of scientific research on plant hormone biosynthesis and signaling during FR has been the investigation of the influence of TFs, such as ripening inhibitor (RIN), colorless non-ripening (CNR), signal-responsive/calmodulin-binding transcription activators (SR/CAMTA), ZFPs, FRUITFULL1/2 (FUL1/2), forever young flower (FYFL), and NAC [1, 3, 10, 21–24]. Although a large number of TFs are involved in the regulation of phytohormones during FR, there are only a few reports that focus on the regulation of GA in this process. In higher plants, GA is a phytohormone of vital importance that belongs to the tetracyclic diterpenoid class and is present ubiquitously. It functions in different phases of the plant’s life cycle, such as seed germination, expansion of leaves, blossoming, setting of fruits, and their subsequent growth [25, 26]. Hence, numerous studies have investigated the effects of GA on seed development and fruit growth [27]. It has been demonstrated that the utilization of GA can improve the firmness of tomato fruits by augmenting the concentrations of ascorbic acid, soluble solids, and fruit weight [28, 29]. Research has shown that the suppression of the levels of GA through overexpression of the GA catabolism gene *SlGA2ox1* in tomato fruit tissues may result in early ripening, suggesting a negative role of GAs in the tomato FR process [30]. Moreover, a bHLH family TF called SlPRE2 has been identified as a negative regulator of chlorophyll (Chl) and Car contents during tomato FR. It mediates the GA pathway and plays a significant role in tomato fruit development [31, 32]. GA synthesis mainly occurs in plant stems, roots, seeds, and fruits. The biosynthetic pathway of GA primarily starts with the cyclization of the C20 precursor geranylgeranyl diphosphate (GGPP), which is converted to kaurine by cobazil diphosphate synthase (CPS) and ent-kaurene synthase (KS). Subsequently, various enzymes act on kaurine to generate GAs (GA_1_, GA_3_, and GA_4_) with diverse biological activities [33]. Endogenous GA levels in plants are maintained through positive/negative feedback regulation of GA metabolism, primarily by controlling the activity of critical enzymes involved in GA synthesis [34–36]. When GA combines with GID1, the soluble receptor for *Gibberellin-insensitive dwarf1* (GID1), it enhances GID1’s affinity toward DELLA, thus initializing the creation of a complex known as GID1-GA-DELLA. The identification of GID1 was first reported in rice [37]. The tomato genome contains three GA receptors, namely, SlGID1a, SlGID1b1, and SlGID1b2, which are believed to mediate specific GA responses [38]. The DELLA protein plays a crucial role in maintaining GA balance and primarily acts as a repressor of gene transcription, inhibiting plant growth and development [39]. It also inhibits the signal transduction process of various plant hormones, including IAA, ETH, and JA [40, 41]. The study’s findings revealed an inverse relationship between the content of GA and DELLA protein in *Arabidopsis*. When the amount of GA is at a low level (there is a high content of the DELLA protein), the up-regulation of GA synthesis genes, specifically *GA20ox* and *GA3ox*, occurs. Nevertheless, the situation is reversed when plants are exposed to exogenous GA treatment [34, 35]. Furthermore, the GA decomposition gene *GA20ox* plays a role in feedback mediation to maintain the dynamic balance of GA. Although studies have reported that certain TFs may mediate the regulation of GA in FR, the detailed regulatory mechanism, especially for NAC TFs, remains unknown.

In our study, we discovered that a NAC TF named SlNAP1 positively regulates tomato FR by directly binding to the promoters of two critical genes in the GA metabolic pathway. The interaction results in the activation of their expression. Our discoveries provide an understanding of the function of NAC TFs and enhance our comprehension of the mechanisms underlying GA production. This investigation enhances our understanding of the regulatory network that controls GA synthesis in tomato FR.

## Materials and methods

### Plant materials and growth conditions

The experimental setup involved the cultivation of transgenic lines and wild-type (WT) tomatoes (*S*. *lycopersicum* cv. Micro-Tom) in a growth chamber. The conditions of the growth chamber consisted of a photoperiod of 16/8 h (light/dark), air temperature of 25/18 ℃ (day/night), relative humidity of 80%, and light intensity of 200 µmol m^−2^ s^−1^. To determine the different ripening stages, flowers were tagged on the basis of the time of anthesis and FR stages were recorded as days post-anthesis (DPA). Both WT and transgenic lines exhibited the following ripening stages in their fruits: mature green (MG; 35 DPA), breaker (Br; fruit exhibiting the first signs of ripening-related color change from green to yellow), 4 days after breaker (Br + 4), and 7 days after breaker (Br + 7). In transient expression experiments, tobacco (*Nicotiana benthamiana*) aged 4–6 weeks was placed in a growth chamber under 25 ℃ light for 16/8 h (light/dark).

### Colorimetric evaluation of tomato fruit

A CM-5 colorimeter was utilized to assess the color of tomato fruits at different stages, namely MG, Br, Br + 4, and Br + 7. In this study, a total of 20 tomato fruits with identical size and color were selected. To measure the redness (a*) and yellowness (b*) of the fruits, three random points were selected along the periphery of each fruit, and the colorimeter was employed.

### Multisequence alignment and phylogenetic analysis

The protein sequences of *Arabidopsis* and tomato were acquired from two databases, namely, the *Arabidopsis* Information Resource (TAIR) and the Sol Genomics Network (SGN). DNAMAN software was utilized to perform protein multiple sequence alignment. The phylogenetic tree was constructed using MEGA-X software and neighbor-joining (NJ) method, and the bootstrap repeat value was set to 1000.

### Total RNA isolation and quantitative real-time polymerase chain reaction (qRT-PCR) analysis

The fruits underwent harvesting at different stages, including the MG, Br, Br + 4, and Br + 7 stages. To extract total RNA from roots, stems, leaves, flowers, and fruits, TRIzol reagent (Vazyme, Nanjing, China) was utilized. Frozen roots, stems, leaves, flowers, or fruits were pulverized into powder using liquid nitrogen. Subsequently, 1 mL of TRIzol was added and hatched at 4 ℃ for 10 min. A 200 μL portion of chloroform was then introduced and continued incubation for 5 min. The mixture was centrifuged at 16,000 *g* at 4 °C for 15 min, leading to the collection of the resultant supernatant. An equal volume of isopropyl alcohol was added, followed by incubation at – 20 ℃ for over 1 h. Finally, the supernatant was collected and transferred to an adsorption column, which was washed with 75% ethanol. The precipitate, dissolved in RNase-free ddH_2_O, was referred to as RNA. To carry out cDNA synthesis, we utilized the FastQuant first strand cDNA synthesis kit (Tiangen, Beijing, China) following the instructions provided by the manufacturer. PCR reaction conditions: 95 °C for 30 s, 95 °C for 5 s, 60 °C for 30 s for 40 cycles; 4 °C, ∞. To serve as an internal control, we employed the *Slactin* gene (Solyc03g078400). To determine the relative expression values, we applied the 2^−△△Ct^ method [42]. Every experiment was reproduced three times biologically, with each replicate including three technical duplications. All primers used for qRT-PCR were listed in Additional file 1: Table S2.

### Gibberellin (GA) and paclobutrazol (PAC) treatments

The study conducted by Zhang et al. [43] served as a reference for the utilization of GA and GA synthesis inhibitor PAC in this experiment. Following the acquisition of tomato fruits, ones with identical sizes were chosen and classified into three separate groups. GA and PAC were dissolved using 6% absolute ethanol and mixed with distilled water to create 10 mM GA and 1 mM PAC solutions, respectively. Subsequently, medical syringes were employed to inject 25 μL of 10 mM GA (group 1), 1 mM PAC (group 2), and 6% anhydrous ethanol (group 3, control group). To ensure accuracy, each treatment was replicated three times. For each replication, 20 tomato fruits were utilized. The fruits were collected after 3 days of treatment and promptly frozen in liquid nitrogen after being stored at − 80 °C for further analysis.

### Virus induced gene silencing (VIGS)

To produce the constructs needed for VIGS, we cloned a particular 200–300 bp segment of SlNAP1 into the pTRV2 vector using primers specifically designed for the gene (Additional file 1: Table S1). Plasmids pTRV1, pTRV2, and pTRV2-SlNAP1 were transformed with *A*. *tumefaciens* GV3101. We then injected the mixture of *A*. *tumefaciens* pTRV2-SlNAP1 (pTRV1:pTRV2-SlNAP1 = 1:1, *v*/*v*) and a control mixture (pTRV1:pTRV2 = 1:1, *v*/*v*) into undamaged and similar size tomato fruits at the MG and Br stages using a needleless syringe with a 1 mL capacity. We performed the infiltration on ten fruits for each VIGS construct. Following infiltration, the tomato fruits were positioned in a growth chamber, kept in darkness, and maintained at a temperature of 25 °C for 3 days. After 3 days postinfiltration, we collected pericarp tissue that exhibited significant inhibition of ripening. The tissue samples collected were promptly frozen in liquid nitrogen and stored at −80 °C until they were required for subsequent utilization. The efficacy of SlNAP1 in hindering gene expression was evaluated through qRT-PCR. Simultaneously, photos at the third day were taken.

### Chlorophyll (Chl) and carotenoids (Car) content measurement

The quantification of total Chl was carried out spectrophotometrically using a previously established formula [44] after extraction with 80% acetone. On the other hand, the quantification of total Car involved the extraction of the compound with 90% ethanol and its subsequent determination spectrophotometrically using a formula described earlier [45].

### Generation of CRISPR/Cas9-SlNAP1 transgenic tomato plants

The selection of two sgRNAs targeting the SlNAP1 gene was performed using CRISPR-P (http://cbi.hzau.edu.cn/crispr/). Additional file 1: Table S3 provides a list of the primers employed in this section. The PCR amplification program consisted of the following parameters: 98 ℃ for 2 min; 95 °C for 10 s; 55 °C for 10 s; 68 °C for 10 s for 35 cycles; and 72 °C for 2 min. Two rounds of PCR amplification were carried out and the resulting sgRNA expression cassettes were simultaneously cloned into the PHEE401 vector. Verification of the PHEE401-SlNAP1 vectors was conducted through sequencing and checking, and transformation into *A*. *tumefaciens* strain GV3101. Subsequently, the vectors were transformed into the “Micro-Tom” tomato cultivar using the method described by Wang et al. [46]. The analysis on *slnap1* mutants carried out on homozygous T0 lines was achieved by extracting DNA from transgenic plants using the fast plant DNAZol reagent (ZenPio, Hangzhou, China) and employing specific primers listed in Additional file 1: Table S3.

### DNA extraction

The DNA extraction kit and DNeasy Plant Mini Kit (Qiagen, Hilden, Germany) were utilized to extract total genomic DNA from tomato leaves following the provided instructions. Further analysis of promoter experiments was conducted using the extracted DNA.

### Determination of endogenous ethylene (ETH) content

The fruits in WT, *slnap1-5*, and *slnap1-16* mutants were randomly selected at the MG, Br, Br + 4, and Br + 7 stages. Then, the fruits were left at room temperature for 2 h to remove the effects of mechanical damage. The fruits were weighted and sealed in a desiccative airtight container at room temperature for additional 10 h. Then, 200 μL of headspace gas from each container was collected using a gas-tight hypodermic syringe and injected into a gas chromatograph (GC-17A, Shimadzu, Kyoto, Japan) immediately for ETH concentration measurement [47].

### Car extraction and high-performance liquid chromatography (HPLC)

The process began by grinding 0.5 g of tomato fruits into a pulpy consistency. Subsequently, a mixture of petroleum ether and acetone (in a 2:1 ratio, *v*/*v*) was introduced to facilitate extraction under ultrasonic conditions. This extraction process was repeated several times until the remaining residue became colorless. The collected extracts were combined into the separating funnel. Through the use of the separating funnel, the organic and aqueous phases were effectively separated, and the upper organic phase was subsequently transferred to a round bottom flask. To eliminate impurities, the extract was subjected to rotational evaporation at a temperature below 45 °C. Subsequently, 25 mL of acetonitrile:dichloromethane:methanol (in a ratio of 55:20:25) was employed to dissolve any remaining impurity solvent. Finally, the resulting solution was filtered using a 0.22 μm membrane and subjected to HPLC analysis using the Waters liquid chromatography system. The HPLCC18 column, with dimensions of 250 mm × 4.6 mm and a particle size of 5 μm, served as the stationary phase. The column temperature was maintained at 25 °C while the flow rate was set at 1.2 mL min^−1^. The mobile phase was a mixture of ethyl eye, dichloromethane, and methanol (ethanol: dichloromethane: methanol = 55:20:25). For the analysis, an injection volume of 10 µL was used, and a detection wavelength of 450 nm was selected.

### Measurement of endogenous gibberellin (GA_3_) content

Extraction of samples involved the use of 5 mL of 80% methanol with a chromatographic method, which had been precooled. These samples were then left to soak overnight at a temperature of 4 °C. Afterwards, the obtained extract was subjected to centrifugation at a speed of 4000 rpm for a duration of 15 min. The resulting residue was then treated with 2.5 mL of 80% methanol for a period of 1 h, followed by another round of centrifugation. The resulting supernatant was combined and subsequently prepared using 80% methanol to reach a final volume of 10 mL. Then, 2 mL of the mixture was subjected to rotation at a speed of 1300 rpm and a temperature of 38 °C for a total of 4 h until complete drying was achieved. Finally, the mixture was resuspended and filtered using 2 mL of 50% methanol with a chromatographic approach. The determination of GA_3_ content was carried out via ultrafast liquid chromatography using the Waters Acquity ARC 600-2998 four-step system, which involved the use of Symmetry-C18 (4.6 mm 250 mm, 5 μm) as the stationary phase. The mobile phase C consisted of 100% methanol with a chromatographic quality, while mobile phase D was composed of 0.1% phosphoric acid of chromatographic purity. Injections of 10 μL were made, and the flow rate was set to 1.0 mL min^−1^. Detection of GA_3_ content took place at a wavelength of 210 nm, while the column temperature was maintained at 30 °C.

### Yeast one-hybrid (Y1H) assay

The assay of Y1H was carried out on the basis of the Matchmaker Gold Yeast One Hybrid System protocol (Clontech, CA, USA). To construct a prey vector, the CDS of SlNAP1 was amplified and merged into pGADT7. The promoter sequences of *SlGA2ox1* and *SlGA2ox5*, covering 1500 bp, were obtained from NCBI databases (https://www.ncbi.nlm.nih.gov/) and merged into pAbAi to form a bait vector. The linearized pAbAi-proSlGA2ox1 and pAbAi-proSlGA2ox5 plasmids were transformed into Y1H Gold yeast strains. Screening for minimal inhibitory concentration of Aureobasidin A (AbA) was performed to avoid any self-activation instances. The bait yeast strains were transformed with the pGADT7-SlNAP1 vector and cultured on SD medium lacking Leu (SD/-Leu) at 30 °C for 2–3 days, both with and without AbA. A control was established using the pAbAi and pGADT7 plasmids. These experiments were repeated three times, yielding similar results, and a representative image is provided.

### Dual-luciferase transient expression assay

To assess the binding activity of SlNAP1 to the promoters of *SlGA2ox1* and *SlGA2ox5*, we first cloned the CDS of SlNAP1 into the pGreenII 62-SK vector as the effector vector. For the reporter vector, we cloned approximately 1.5-kb-long promoters of *SlGA2ox1* and *SlGA2ox5* genes into the pGreenII 0800-LUC vector [48]. Subsequently, we transferred the resulting plasmids into *A*. *tumefaciens* GV3101 and injected them into tobacco leaves using a needleless syringe (1 mL), following the protocol established by Hellens et al. [48]. To determine the luciferase activity of LUC and REN, we utilized the Dual-Luciferase Assay Kit (Promega, MA, USA).

### Bimolecular fluorescence complementation (BiFC) assay

The pCAMBIA1300-YFPN or pCAMBIA1300-YFPC vector was used to clone SlNAP1 and SlGID1 full-length CDS sequences, excluding stop codons. The constructs were transferred to *A*. *tumefaciens* strain GV3101, following the protocols for *A*. *tumefaciens*-mediated transient expression in tobacco leaves aged 4 weeks [49]. After incubation at 22 °C for 24–48 h, the confocal laser scanning microscope (Zeiss LSM 800, Oberkochen, Germany) was used to observe YFP fluorescence. YFP excitation occurred at a wavelength of 514 nm, and emission at 534 nm. Additional file 1: Table S1 provides the list of primers used for vector construction. The experiments were repeated three times and produced consistent results, along with a representative image.

### Yeast-two-hybrid (Y2H) interaction study

To verify the interaction between SlNAP1 and SlGID1, we subcloned the CDS of SlNAP1 and SlGID1 into either the pGBKT7 or pGADT7 vector. The vectors were coconverted to yeast Y2H using the lithium acetate method and grew on DDO medium (minimum medium double drops, SD medium with -Leu/-Trp added) for 3 days according to the manufacturer’s protocol (Clontech, San Francisco, USA). Transformed colonies were plated onto the QDO (minimal media quadruple dropouts, SD medium supplemented with -Leu/-Trp/-Ade/-His) medium to test the possible protein–protein interaction. The ability of yeast cells to grow on the QDO medium was scored as a positive interaction. The experiments were repeated three times.

### Firefly luciferase complementation experiment

The firefly luciferase complementation experiment was primarily conducted by following Chen et al.’s [50] method. The gene sequences of SlNAP1 (without a stop codon) were amplified and cloned into the pCAMBIA1300-nLUC vector, while the CDS of SlGID1 were cloned into the pCAMBIA1300-cLUC vector. The constructs were transformed with *A*. *tumefaciens* strain GV3101. Infiltration of the *A*. *tumefaciens* GV3101 mixture (pCAMBIA1300-nLUC: pCAMBIA1300-cLUC = 1:1, *v*/*v*) into tobacco leaves was achieved using a needleless syringe with 1 mL capacity. Following a 3-day incubation in a greenhouse, luminescence was observed using optical in vivo imaging (PlantView 100, Guangzhou, China) after applying a fluorescent substrate to the back of the leaf. Furthermore, to investigate the impact of GA on the interaction between SlNAP1 and SlGID1, solutions of 6% absolute ethanol, 10 mM GA, or 1 mM PAC were sprayed after the infiltration of the bacterial solutions. Similarly, luminescence was observed under various treatment conditions (Bars = 25 μm).

### Statistical analysis

The data in this study were obtained from at least three biological replicates and were shown as means ± standard deviation (SD). Pairwise comparison was performed using Student’s *t* test. The figures depict significant differences with a single asterisk (*) for *P* < 0.05 and with double asterisks (**) for *P* < 0.01.

## Results

### SlNAP1 is a typical NAC transcript factor

The *SlNAP1* gene (Solyc05g007770.2.1) is located on chromosome 5, which consists of two introns and three exons. SlNAP1, with a full-length CDS of 849 bp, encodes the SlNAP1 protein comprising 282 amino acids. Analysis of multiple sequence alignments indicated that SlNAP1 is a member of the NAC TF family and possesses the characteristic N-terminal NAC domain structure. This structure is further divided into five subdomains (A–E), while the C-terminus contains an unconserved transcriptional activation domain (Additional file 1: Fig. S1).

### SlNAP1 gene is expressed throughout tomato FR stage

The evolutionary relationship between SlNAP1 and other NAC TFs in *Arabidopsis* and tomato were examined using phylogenetic analysis. It was observed that the SlNAP1 protein exhibited a strong association with NAC TFs that are involved in FR, namely SlNAC9, SlNAC4, SlNAC1, SlNAM1, SlNAC-NOR, and SlNOR-like1 (Additional file 1: Fig. S2). These findings suggest that SlNAP1 may hold significant significance in the process of tomato FR.

**Fig. 1:**
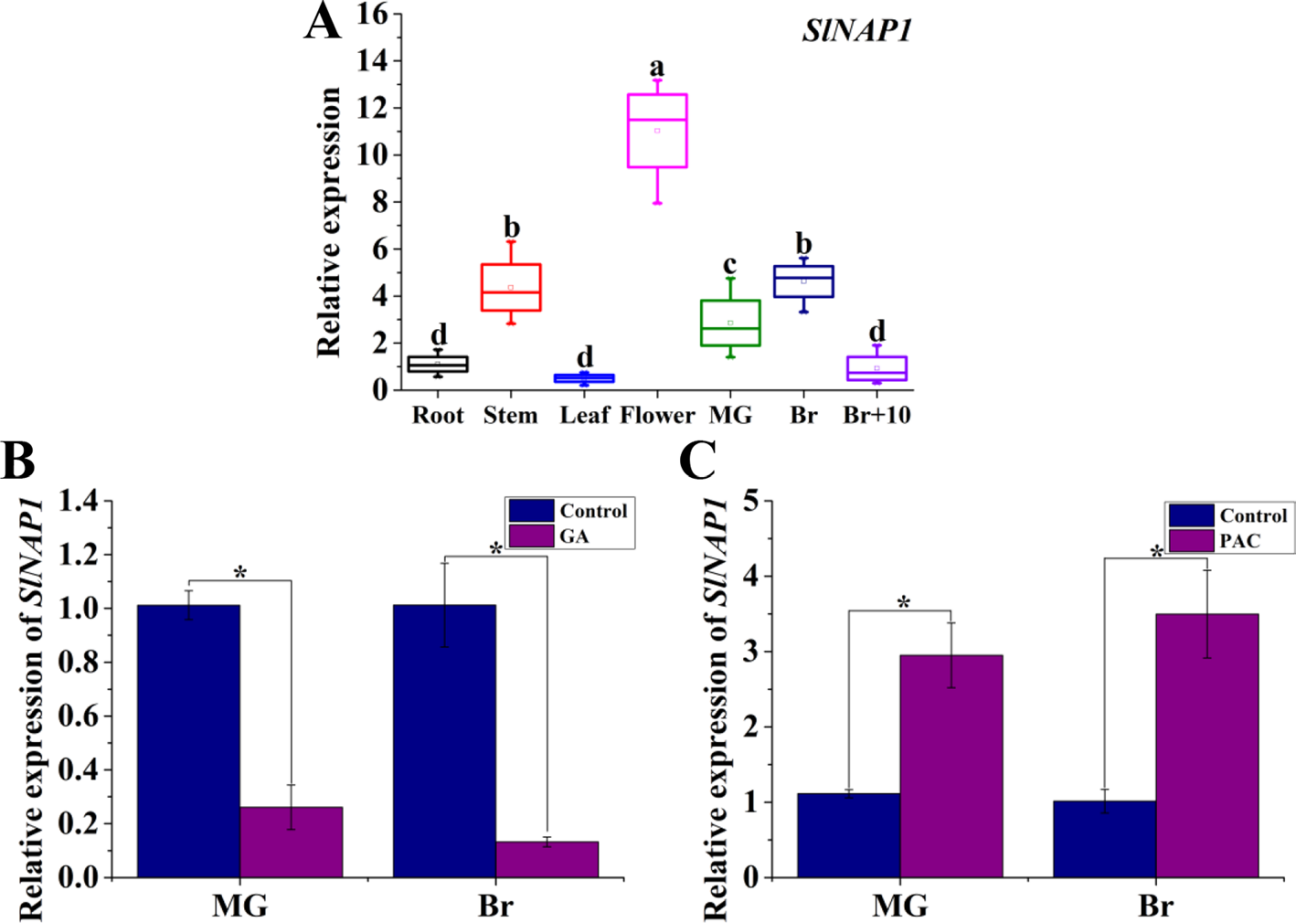
SlNAP1 transcripts in different organs of tomato plants and after the treatment of wild-type (WT) fruit with gibberellin (GA) and paclobutrazol (PAC). **A** Quantitative real-time polymerase chain reaction (qRT-PCR) of SlNAP1 in different tomato organs (root, stem, leaf, and flower) and fruit ripening stages (MG, Br, and Br + 10). **B** Transcriptional level of SlNAP1 in WT fruit after treatment with GA. **C** Transcriptional level of *SlNAP1* in WT fruit after treatment PAC. The Slactin gene was used as the internal control. Bars indicate mean ± SD of three independent replicates. Asterisks indicate significant differences determined by Student’s *t*-test (*P < 0.05). *qRT-PCR* quantitative real-time PCR, *MG*, mature green, *Br* breaker, *Br + 10* 10 days after the breaker, *SD* standard error

To analyze the expression patterns of *SlNAP1* in tomato, a diverse collection of tomato tissues including root, stem, leaf, and flower were gathered. Additionally, tomato fruits at distinct ripening stages, namely MG, Br, and 10 d post-breaker (Br + 10), were also obtained for examination. Subsequently, the levels of *SlNAP1* transcripts were assessed using qRT-PCR. *SlNAP1* was found to be expressed across all tissues, with particularly high expression levels observed in the stem and flower (Fig. 1A). Furthermore, during tomato FR, there was a significant induction of *SlNAP1* transcript levels. Initially, they increased, reached a peak at the Br stage, and subsequently declined (Fig. 1A). These findings suggest that SlNAP1 may exhibit a distinct pattern of expression associated with the ripening process.

In our study, it was observed that exogenous GA exerted a significant repression on the transcripts of SlNAP1 in fruits at the MG and Br stages, whereas PAC, an inhibitor of GA perception, induced the expression of *SlNAP1* (Fig. 1B and C). The observed expression pattern of SlNAP1 in tomato fruit led us to hypothesize its involvement in the FR process, specifically through the GA pathway. However, to confirm this assumption, a comparative analysis of the ripening process in WT, TRV-SlNAP1, and SlNAP1-edited tomato fruits is necessary.

### SlNAP1 gene is related to FR

To ascertain whether there is a potential association between SlNAP1 and the process of FR, we obtained a striking inhibition of ripening in VIGS fruits infected with a TRV-*SlNAP1* construct. Instead of displaying a uniform green or orange phenotype as observed in the control fruits at MG or Br, the infected fruits showcased distinct mottled green and orange areas that were clearly demarcated by a distinct border (Additional file 1: Fig. S3A). Additionally, the qRT-PCR analysis revealed a substantial decrease in the levels of *SlNAP1* transcripts in the green or orange sections of the TRV-*SlNAP1*-infected fruits compared with those in the corresponding sections of the control fruits infected solely with TRV (Additional file 1: Fig. S3B), indicating a correlation between the silencing of the *SlNAP1* gene and the uneven color phenotype. Furthermore, the TRV-*SlNAP1* fruits exhibited significant reductions in both the a value and b value at the MG and Br stages when compared with the TRV-control fruits (Additional file 1: Figs. S3C and D). This suggests that the absence of SlNAP1 influenced the color development of the fruits. Specifically, while the accumulation of Chl was observed at the MG and Br stages, Car underwent degradation solely at the Br stage in the TRV-*SlNAP1* fruits (Additional file 1: Figs. S3E and F). Moreover, the expression levels of several ripening-associated genes, including *NOR*, *RIN*, *E4*, and *E8*, were noticeably lower in the TRV-*SlNAP1* fruits in comparison with the TRV-control fruits (Additional file 1: Fig. S3G). This provides further evidence that SlNAP1 likely plays a role in the regulation of FR.

**Fig. 2:**
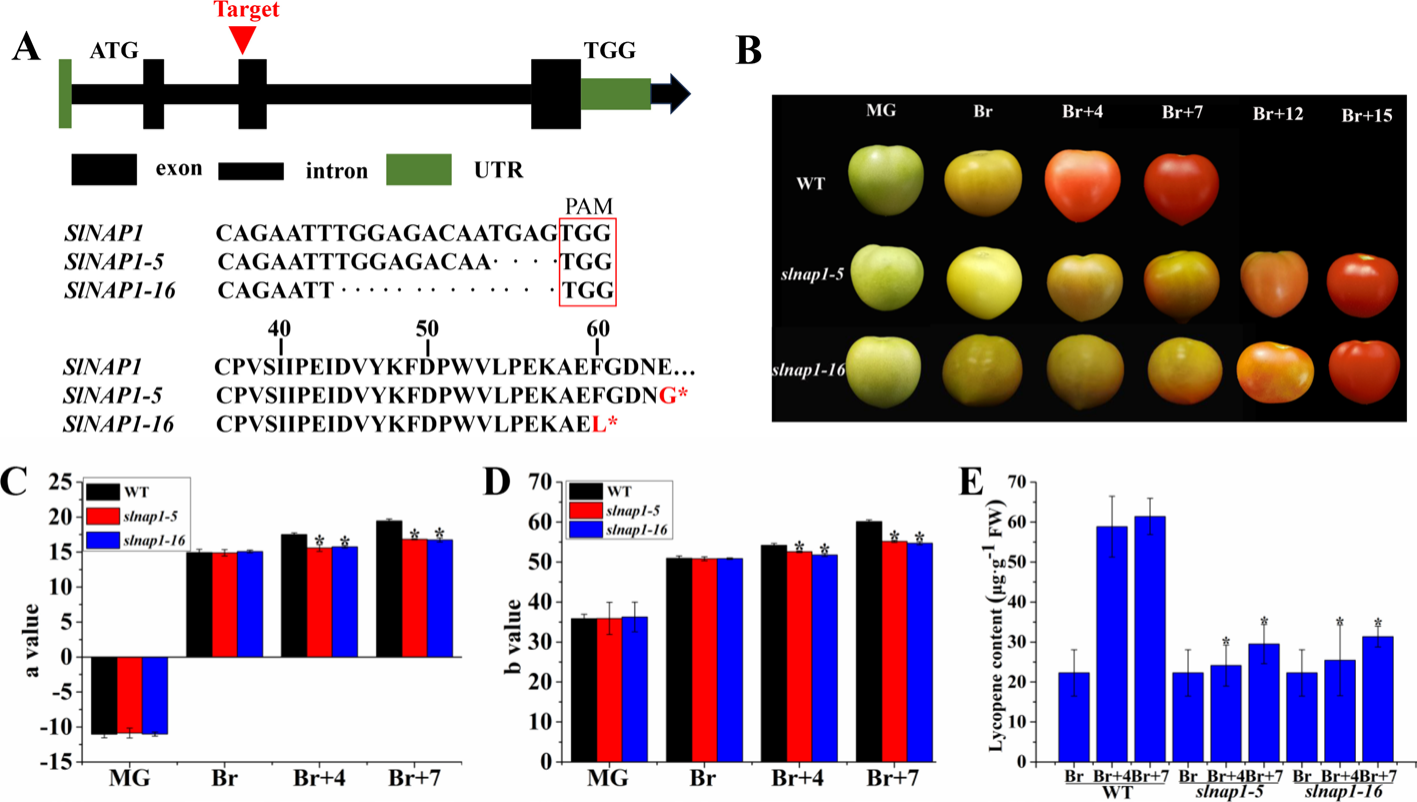
Tomato fruit ripening phenotype of wild-type (WT) and CR-SlNAP1. **A** Gene editing analysis of *slnap1-5* and *slnap1-16* homozygous mutants. **B** Phenotype of tomato fruit in WT, slnap1-5, and slnap1-16. WT, Wild-type. **C** Value of tomato fruit in WT, slnap1-5, and slnap1-16 at MG, Br, Br + 4 and Br + 7 stages. **D** *b* Value of tomato fruit in WT, slnap1-5, and slnap1-16 at MG, Br, Br + 4, and Br + 7 stages. **E** Lycopene content of tomato fruit in WT, slnap1-5, and slnap1-16 at MG, Br, Br + 4, and Br + 7 stages. Bars represent mean ± SD of three biological replicates. Asterisks indicate significant differences determined by Student’s *t*-test (*P < 0.05). *MG* mature green, *Br* breaker, *Br + 4* 4 days after the breaker, *Br + 7* 7 days after the breaker, *SD* standard error

To examine the potential role of SlNAP1 in the regulation of tomato FR, we utilized CRISPR/Cas9-mediated gene editing to create a tomato SlNAP1 knock-out. Two gene editing lines, namely *SlNAP1-5* and *SlNAP1-16*, were generated, both of which exhibited homozygous deletions in the second exon, removing 4 and 11 base pairs, leading to a premature termination at the 64th (*SlNAP1-5*) and 60th (*SlNAP1-16*) amino acids of the SlNAP1 protein, respectively (Fig. 2A).

To investigate the impact of gene manipulation on tomato FR, we documented the phenotypic changes of 30 representative plants for each line throughout the ripening process. Consequently, a distinct disparity in FR in nearly all the fruits was observed when comparing CR-SlNAP1 and WT plants (Fig. 2B). Phenotypic analysis showed that the *slnap1-5* and *slnap1-16* fruits exhibited a noticeable delay in altering their color compared with WT fruits. While WT fruits reached the red stage, the *slnap1-5* and *slnap1-16* fruits were still at the Br, Br + 4, and Br + 7 stages (Fig. 2B). We also observed that *slnap1* mutant tomato fruit entered red ripe stage at Br + 15, which was 8 days later than WT (Fig. 2B). Our collective findings indicate that SlNAP1 acts as a positive regulator in the tomato FR process. Assessment was conducted on several crucial parameters associated with FR, namely ETH content, fruit saturation levels (a value and b value), lycopene, Chl and Car content, and the gene expression of Chl degradation and Car biosynthesis. The ETH content increased gradually during the ripening process of WT, the *slnap1-5* and *slnap1-16* fruits, and was consistently higher in WT fruits than in *slnap1-5* and *slnap1-16* fruits (Additional file 1: Fig. S4). Specifically, the ETH content in the *slnap1* mutant was 1.4-fold lower that in WT at Br + 4 and Br + 7 stages. The changes in ETH content were consistent to the expression patterns of ETH synthesis genes *SlACO1*, *SlACO3*, *SlACS2*, and *SlACS4*. The expression levels of ETH synthesis genes were decreased compared with WT at MG, Br, Br + 4, and Br + 7 stages, suggesting inhibited ETH production in *slnap1-5* and *slnap1-16* fruits (Additional file 1: Fig. S4). Notably, the a value and b value of *slnap1-5* and *slnap1-16* fruits exhibited significant decreases at Br + 4 and Br + 7 stages in comparison with WT fruits (Fig. 2C and D). It was observed that *slnap1-16* fruits exhibited a delay in Chl degradation during MG stage. Furthermore, *slnap1-5* and *slnap1-16* fruits notably hindered Car biosynthesis at Br, Br + 4, and Br + 7 stages (Additional file 1: Fig. S5). At the Br stage, the lycopene content in *slnap1-5* and *slnap1-16* fruits experienced a substantial decrease (in Fig. 2E). Additionally, the silencing of *SlNAP1* resulted in reduced expression levels of degradation-related genes, such as *SlNYC1*, *SlPPH*, *SlRCCR*, *SlSGR1*, and *SlPAO*, as well as Car biosynthesis-related genes, including *SlPSY1*, *SlPSY2*, *SlCPPS*, *SlIDS*, *SlLCYB1*, *SlLCYB2*, *SlLCYE*, *SlCYHB1*, *SlCYHB2*, *SlVED*, and *SlZFP*, with the exception of *SlPSY3* (Additional file 1: Figs. S6 and S7; [51–53]). These findings suggest that SlNAP1 plays a critical role as an activator in facilitating the color transformation process during tomato FR.

### SlNAP1 inhibits endogenous GA_3_ degradation in ripening fruits

**Fig. 3:**
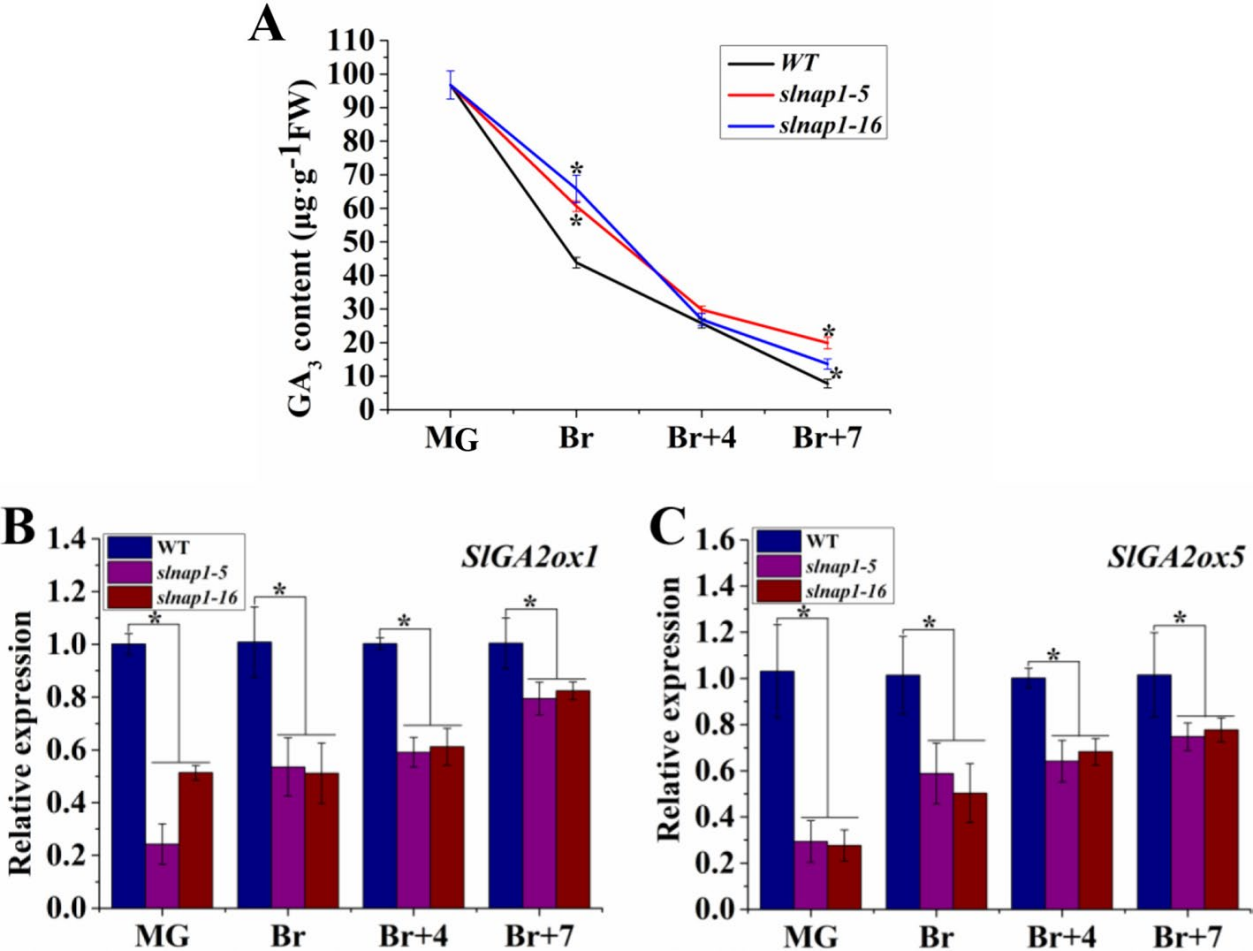
Endogenous gibberellin (GA_3_) content and related genes expression in wild-type (WT), *slnap1-5*, and *slnap1-16* tomato fruits at mature green (MG), breaker (Br), 4 days after breaker (Br + 4), and 7 days after breaker (Br + 7) stages. **A** GA3 content in WT, *slnap1-5*, and *slnap1-16* tomato fruits at MG, Br, Br + 4, and Br + 7 stages; **B** relative expression of SlGA2ox1 gene in WT, slnap1-5, and slnap1-16 tomato fruits at MG, Br, Br + 4, and Br + 7 stages; **C** relative expression of SlGA2ox5 gene in WT, *slnap1-5*, and *slnap1-16* tomato fruits at MG, Br, Br + 4, and Br + 7 stages

To investigate the impact of SlNAP1 on the GA_3_ levels during tomato FR, the endogenous GA_3_ content was measured in *slnap1-5*, *slnap1-16*, and WT fruits at different stages, namely MG, Br, Br + 4, and Br + 7. The results showed a noticeable delay in the reduction of endogenous GA_3_ content in *slnap1-5* and *slnap1-16* fruits compared with WT fruits at Br and Br + 7 stages (Fig. 3A). Furthermore, the expression levels of genes related to GA degradation, namely *SlGA2ox1* and *SlGA2ox5*, were significantly decreased in *slnap1-5* and *slnap1-16* fruits at MG, Br, Br + 4, and Br + 7 stages when compared with those in WT fruits (Fig. 3B and C).

### SlNAP1 binds the promoters of *SlGA2ox1* and *SlGA2ox5* to suppress GA production during FR

**Fig. 4:**
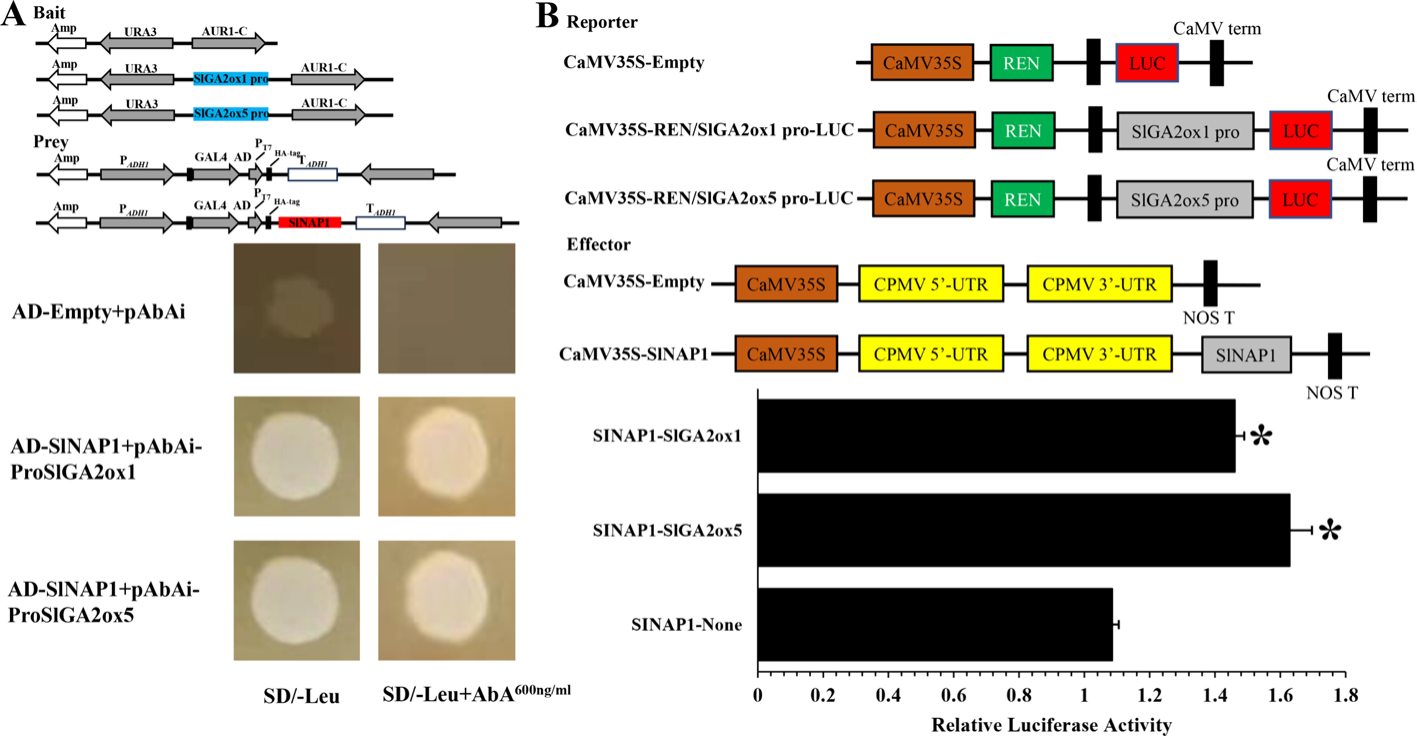
SlNAP1 directly binds to the promoters of *SlGA2ox1* and *SlGA2ox5*. **A** Y1H assays. The CDS of SlNAP1 was fused to the pGADT7 vector, and the promoter fragments of *SlGA2ox1* and *SlGA2ox5* corresponding to the regions −1500 to −1 were fused to pAbAi vector. **B** DLR assays. The CDS of SlNAP1 was cloned into the pGreenII 62-SK vector to generate the SlNAP1-62SK effector. The promoters of SlGA2ox1 and SlGA2ox5 (1500-bp upstream of the start codon) were introduced into the pGreenII 0800-LUC vector to generate the ProSlGA2ox1:LUC and ProSlGA2ox5:LUC, reporter constructs, respectively. The constructs were transformed into *Agrobacterium tumefaciens* strain GV3101. *A*. *tumefaciens* was mixed and coinfiltrated into tobacco leaves for transient expression. A DLR assay system was used to measure the ratio of luminescence of firefly LUC to Renilla LUC. Each value represents the means of six biological replicates. *P < 0.05 (Student’s *t*-test). *Y1H* yeast one-hybrid, *DLR* dual-luciferase reporter, *CDS* coding sequence

The key role of *SlGA2ox1* and *SlGA2ox5* in GA degradation has been demonstrated previously, and we speculated that *SlGA2ox1* and *SlGA2ox5* might be the direct targets of SlNAP1. Y1H was used to test whether SlNAP1 TF binds directly to the promoters of *SlGA2ox1* and *SlGA2ox5* genes in vitro. As shown in Fig. 4A, SlNAP1 binds to the promoters of *SlGA2ox1* and *SlGA2ox5* in vitro. The expression profiles of *SlGA2ox1* and *SlGA2ox5* in *slnap1* mutants indicated that SlNAP1 may positively regulate the transcription of *SlGA2ox1* and *SlGA2ox5* (Fig. 4B and C). To further examine the hypothesis, we conducted dual-luciferase reporter (DLR) assays. The relative LUC/REN ratio in tobacco leaves cotransformed with CaMV35S-SlNAP1 and either CaMV35S-REN/pSlGA2ox1-LUC or CaMV35S-REN/pSlGA2ox5-LUC exhibited a significantly elevated level in comparison with that in leaves cotransformed with CaMV35S-Empty and either CaMV35S-REN/pSlGA2ox1-LUC or CaMV35S-REN/pSlGA2ox5-LUC (Fig. 4B). This suggests that SlNAP1 could bind to and activate the promoters of *SlGA2ox1* and *SlGA2ox5 *in vivo. Consequently, it is plausible to propose that SlNAP1 potentially governs the regulation of tomato FR by modulating the transcriptional activity of two GA degradation genes, namely *SlGA2ox1* and *SlGA2ox5*.

### SlNAP1 interacts with SlGID1

**Fig. 5:**
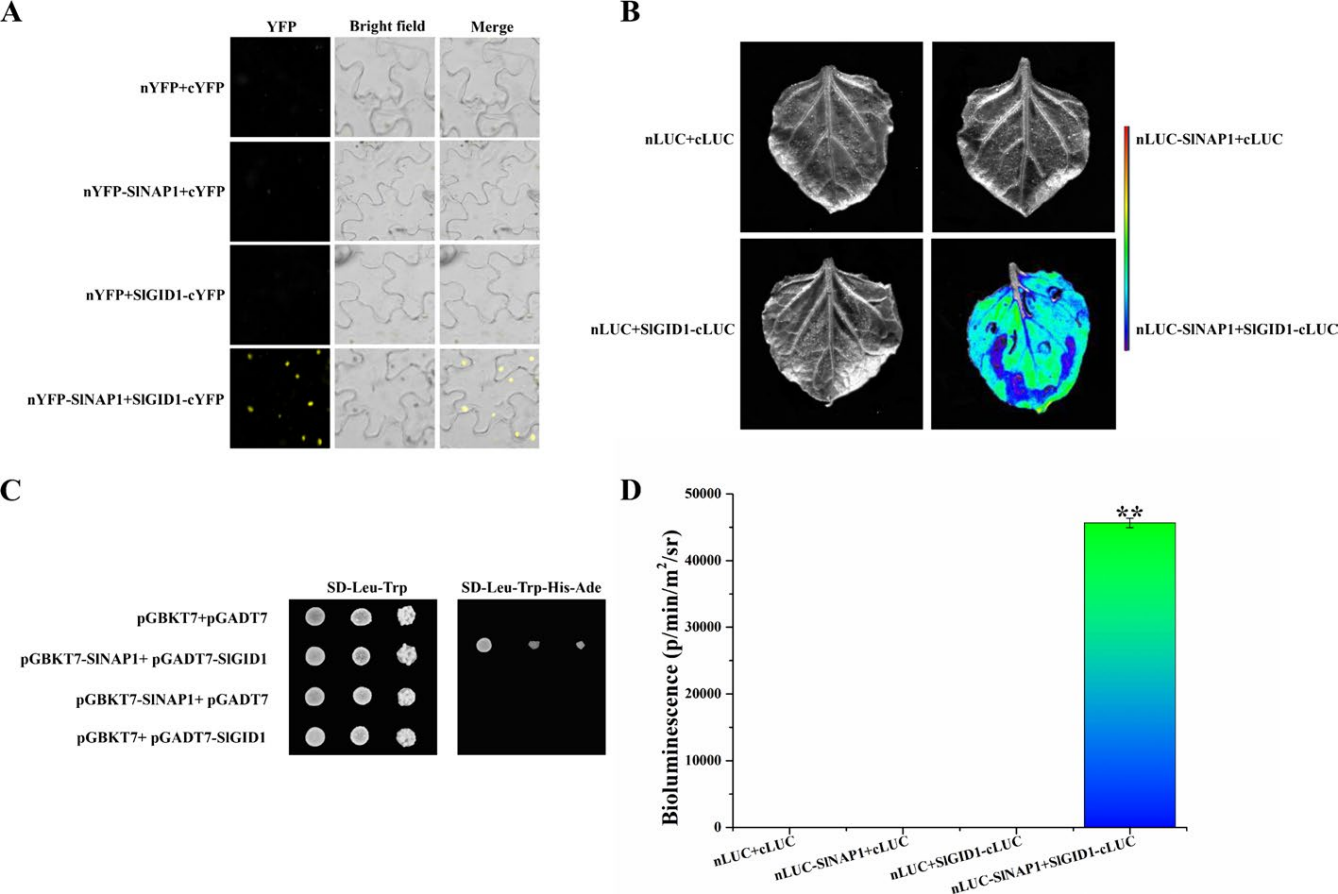
SlNAP1 interacts with SlGID1 in vitro and in vivo. **A** BiFC assays. Full-length SlNAP1 and SlGID1 were fused to the N-terminal part and the C-terminal part of YFP, respectively. Constructs were transformed to *Agrobacterium tumefaciens* strain GV3101, and were then injected into 4-week-old tobacco leaves. The YFP fluorescence was observed under a confocal laser scanning microscope after incubating at 22 °C for 24–48 h. Bars = 50 μm. **B** Y2H assays. The full-length SlGID4 was fused with the activation domain (pGADT7-SlGID4) and the full-length SlNAP1 was fused with the binding domain (pGBK7-SlNAP1). Transformed yeast cells were grown on SD-Leu-Trp, or SD- Leu-Trp-His-Ade media. These experiments were performed three times with similar results, and a representative picture was shown. **C** and **D** LUC assays. CDS of SlNAP1 (with no stop codon) was cloned into pCAMBIA1300-nLUC, and the CDS of SlGID1 was cloned into the pCAMBIA1300-cLUC vector. The constructs were transformed into *Agrobacterium tumefaciens* strain GV3101, and *A*. *tumefaciens* was mixed (1:1, *v*/*v*) and coinfiltrated into tobacco, and luminescence was observed in optical in vivo imaging and was analyzed by PlantView. *BiFC*, bimolecular fluorescence complementation; *DAPI* 4, 6-diamidino-2-phenylindole, *Y2H* yeast two-hybrid, *LUC* Luciferase

To ascertain if there is an association between SlNAP1 and SlGID1 (Solyc01g098390), various techniques were employed, including BiFC and Y2H assays, as well as LUC assays. The in vivo interaction between SlNAP1 and SlGID1 was assessed using the BiFC assay. Tobacco plants were cotransformed with SlNAP1 and SlGID1. By employing a confocal laser scanning microscope (Zeiss LSM 800, Oberkochen, Germany), the BiFC assay successfully verified the presence of an interaction between SlNAP1 and SlGID1 within the nucleus (Fig. 5A).

In yeast, the coexpression of SlGID4 fused to the GAL4 DNA-binding domain (BD) and SlNAP1 fused to the GAL4 DNA-activating domain (AD), and vice versa. As a result, yeast cells demonstrated growth on selective media without leucine, tryptophan, histidine, and adenine. To evaluate the specificity of the interaction, negative controls were implemented. Yeast cells cotransformed with prey plasmids containing SlNAP1 and an empty bait plasmid, along with the reverse combination of SlGID1 and an empty prey plasmid, were used as control samples. The lack of growth on selective medium in these control samples demonstrated an in vitro interaction between SlNAP1 and SlGID1 (Fig. 5B). The LUC assay was utilized to examine the interaction in *vivo* between SlNAP1 and SlGID1. SlNAP1 and SlGID4 were cotransformed into tobacco, and the results showed the interaction between SlNAP1 and SlGID4 in vivo by optical in vivo imaging (Fig. 5C and D). These outcomes provide evidence that SlNAP1 and SlGID1 can physically interact both in vitro and in vivo. Notably, the interaction between SlNAP1 and SlGID1 was intensified when treated with GA, whereas it was weakened with PAC administration (Additional file 1: Fig. S8).

## Discussion

NAC TFs, known as one of the most abundant TF families found exclusively in plants, play crucial roles in diverse physiological processes associated with plant development and stress tolerance [52–56]. Extensive research has confirmed the existence of numerous NAC family members across various plant species, such as *Arabidopsis*, tomato, banana, and so on [1, 57, 58]. Considering the range of stimuli, it is highly likely that the transcriptional regulation of NAC TFs can be influenced, leading to either upregulation or downregulation [59, 60]. The provided information clearly indicates that SlNAP1, as evident from Additional file 1: Fig. S1, is a member of the NAC TF family and possesses a NAC domain that is highly conserved. Several investigations have demonstrated the involvement of multiple NAC TFs in tomato FR [7, 9, 11]. The accumulation of lycopene and ß-carotene exhibited a declining trend in SlNAC4 RNA interference (RNAi) fruit, implying that SlNAC4 plays a crucial role as a positive regulator in the process of tomato ripening [9]. Suppression of SlNAC4 and SlNAC9 led to a decrease in the expression of ETH sensing genes, thereby hindering FR in SlNACs RNAi lines [7]. Similarly, overexpression of SlNAM1 in OE-SlNAM1 lines accelerated FR, while its suppression in CR-SlNAM1 lines resulted in delayed ripening [11]. The phylogenetic analysis presented in this study demonstrates a close relationship between the SlNAP1 protein and SlNAC9, as shown in Additional file 1: Fig. S2, implying that SlNAP1 might also have a significant role in regulating FR.

The expression profiles of SlNAP1 at the flower and Br stages in this investigation imply that *SlNAP1* mainly regulated tomato FR (Fig. 1A). Mottled green and orange patches, separated by a distinct border phenotypically, were observed in the VIGS of *SINAP1*, unlike the uniform green or orange phenotypes seen in WT fruits at the MG, Br, Br + 4, and Br + 7 stages (Additional file 1: Fig. S3A). On the basis of these findings, a noticeable delay in color transition was observed in the silencing SlNAP1 lines (Additional file 1: Fig. S3A). Concurrently, the suppressed SlNAP1 lines significantly augmented Car degradation at the Br stage and Chl accumulation at the MG stage (Additional file 1: Fig. S3E and F). The change in color is a critical phenotypic marker of the ripening process in tomato fruit, primarily attributed to the breakdown of Chl and the accumulation of Car, which contains lycopene [61]. Lycopene, specifically, is the prevailing Car in tomato fruit. The mutational lines of *slnap1-5* and *slnap1-16* displayed ripening defects at the Br stage in the pericarp compared with WT fruits, indicating alterations in pigmentation (Fig. 2B). To investigate why *slnap1-5* and *slnap1-16* failed to entirely turn red, we conducted HPLC analysis of lycopene levels in WT and *slnap1* mutant fruits at Br, Br + 4, and Br + 7 stages. Our results showed a significant reduction in lycopene levels in *slnap1-5* and *slnap1-16* fruits. Similarly, the measurement of lycopene content in *nor-like1* mutant tomato confirmed that the Car content was lower in mutant fruits compared with WT fruits [11]. Phytoene synthase 1 (PSY1) is a critical enzyme of Car biosynthesis in tomato, which is involved in regulating flux during ripening [62]. According to a study conducted by Ma and colleagues [1], it was discovered that the TF SlNAC1 directly binds to the *SIPSY1* promoter, exerting control over the FR process. Our results show a significant downregulation of several key genes involved in Car synthesis, including *SlPSY1*, *SlPSY2*, *SlCPPS*, *SlIDS*, *SlLCYB1*, *SlLCYB2*, *SlLCYE*, *SlCYHB1*, *SlCYHB2*, and *SlVED* in *slnap1-5* and *slnap1-16* fruits. Interestingly, the expression of *SlPSY3* was found to be upregulated (Additional file 1: Fig. S6). This discrepancy may be attributed to the influence of alternative signaling pathways during the ripening progression of fruit. During FR, Chl degradation and the expression changes of many related transcripts are the main physiological processes. It was observed that the expression levels of Chl degradation genes (*SlNYC1*, *SlSGR1*, *SlPAO*, *SlPPH*, and *SlRCCR*) were notably reduced at the MG stage in *slnap1-5* and *slnap1-16* fruits (Additional file 1: Fig. S6). The upregulation of genes linked to Chl breakdown by the SlNAP1 TF is known to facilitate FR in tomatoes (Additional file 1: Fig. S6). On the basis of our findings and existing evidence, it is suggested that NAP1 may mediate Chl degradation and Car accumulation, thus influencing changes in fruit color. The repression of SlNAC4 expression through RNA interference (RNAi) resulted in diminished Chl metabolism and Car accumulation, consequently impeding FR at the Br stage [9]. Conversely, overexpression of SlNAC1 decreased lycopene accumulation and ETH production, indicating that SlNAC1 may serve to restrict these processes, thereby suppressing FR [1]. These observations underscore the intricate nature of the NAC pathway’s involvement in FR. In conclusion, the findings demonstrate the positive regulatory role of SlNAP1 during tomato FR.

In the examination of FR, a complex network of TFs play a crucial role. This process is influenced by internal hormonal signals as well as external environmental cues. The relationship between phytohormones and tomato FR has been extensively examined in recent scientific investigations. One particular phytohormone, ABA, has been found to regulate various aspects of tomato fruit quality. This includes its ability to enhance tomato fruit softness and colors through the enhancement of relevant enzyme activities and gene expressions [63]. The process of fruit softening is further accelerated by the combined actions of ABA and the TF SlNAC4, which operates through an ABA-dependent pathway. Moreover, ABA content alterations, resulting from the suppression of SlNAC4/9, have been found to have an additional impact on FR [64]. MpSNAC67, a NAC TF from banana, was reported to induce the senescence of fruit, which is dependent on SA pathway [65]. Additionally, the expression of SlNAC4 in tomato was effectively blocked by RNAi, resulting in the downregulation of genes associated with ETH production and the prevention of ETH biosynthesis [9]. The acceleration of *Fragaria chiloensis* FR was attributed to JA, which controlled anthocyanin accumulation, altered cell wall structure, and promoted ETH production [66]. Previous research indicated that the overexpression of the tomato BRI1 gene, a receptor protein for BR, enhanced FR and ETH biosynthesis [67]. Conversely, GA inhibited the transcript levels of ETH-related genes, leading to a delay in tomato ripening [68]. Thus, the application of exogenous ABA, JA, and BR treatments on tomato fruits resulted in accelerated FR [69, 70]. Conversely, the treatment of tomato fruits with auxin and GA_3_ caused a delay in the ripening phenotype [71–74]. Notably, our current investigation revealed that exogenous GA treatment significantly suppressed *SlNAP1* transcripts in tomato fruits, while exogenous PAC enhanced their expression (Fig. 2B and C). Analysis of the expression pattern of SlNAP1 in tomato fruits indicated its potential role in the GA signaling pathway-dependent ripening process. To further elucidate the association between SlNAP1 and GA_3_ during tomato FR, we measured the endogenous GA_3_ content in *slnap1-5*, *slnap1-16*, and WT fruits at MG, Br, Br + 4, and Br + 7 stages. Interestingly, *slnap1-5* and *slnap1-16* fruits exhibited a significant decrease in endogenous GA_3_ level during the MG, Br, Br + 4, and Br + 7 stages (Fig. 4A). Hence, it is plausible that the SlNAP1 TF has the ability to alter the intrinsic GA_3_ levels during the ripening process of tomato fruits. When comparing the *slnap1-5* and *slnap1-16* plants with the WT plants, we observed that the vegetative development of the former was relatively slower. Wang et al. [46] reported that the fruit size of transgenic *SlNAP1*-overexpressing plants was smaller compared with that of the WT plants. However, the fruit number per plant and the fruit yield of the *SlNAP1*-overexpressing plants increased significantly compared with those of the WT plants. In our study, the fruit size and fruit yield of the CR-*SlNAP1* mutants had no significant difference with those of the WT plants. Consequently, further investigation into the role of SlNAP1 in GA signaling during FR is imperative. Zhu et al. [75] demonstrated that the bHLH family TF gene *SlPRE2*, which is inducible by GA, exhibited significant expression levels in immature green tomato fruits. Additionally, they showed that the silencing of SlPRE2 led to an upregulation of genes associated with GA metabolism (*SlGA2ox1*, *SlGA2ox2*, *SlGA20ox1*) in immature green fruits. Furthermore, *OsWOX3A* was identified as a GA-responsive gene that played a crucial role in the negative feedback regulation within the GA biosynthesis pathway to maintain GA_3_ homeostasis and modify the characteristics of rice plants [76]. Similarly, through its influence on the expression levels of *OsGA2ox1* and *OsGA2ox6*, the rice TF OsMADS57 can potentially control plant height by modulating GA_3_ catabolism [77]. The study also revealed that at various stages, including MG, Br, Br + 4, and Br + 7, the downregulation of GA degradation genes, such as *SlGA2ox1* and *SlGA2ox5*, was significantly evident in *slnap1-5* and *slnap1-16* fruits (Fig. 3B and C). This indicates that the transcription of the GA_3_ degradation genes *SlGA2ox1* and *SlGA2ox5* might be regulated by the SlNAP1 TF, potentially influencing the ripening process of tomato fruit. Additionally, the SlNAP1 protein was found to directly bind to the promoters of *SlGA2ox1* and *SlGA2ox5* genes in vitro, positively regulating their transcription, as confirmed by Y1H and DLR experiments (Fig. 4). These findings suggest that SlNAP1 may directly target the transcription of *SlGA2ox1* and *SlGA2ox5* to modulate GA homeostasis during the ripening of tomato fruit. Thus, the *SlNAP1* gene located in GA signal transduction pathways is involved in the feedback regulation of the GA biosynthesis to respond the GA signal to further mediate tomato FR.

The pivotal role of GA receptor proteins in the control of plant growth and development is closely associated with the function of GA. Various reports have highlighted the significance of GA receptor proteins in processes, such as fruit setting [78], seed dormancy [79], and plant growth [80]. A recent research has indicated that *Arabidopsis* exhibits three GA receptors, namely GID1A, GID1B, and GID1C [81], whereas rice possesses only one GA receptor, GID1 [82]. The tomato genome encodes a single DELLA protein *PROCERA* (*PRO*) and three receptor genes, *GID1a*, *GID1b1*, and *GID1b2* [83]. To execute its biological actions, GA binds to its receptor protein GID1. Subsequently, DELLA interacts with the GID1-GA complex to form the GID1-GA-DELLA complex, thereby regulating gene expression [84]. Hence, it remains uncertain whether GID1 proteins can inhibit GA production controlled by SlNAP1. Here, SlNAP1 and GID1 could physically interact with each other in vitro and in vivo (Fig. 5). Furthermore, our investigations have unveiled that while exogenous PAC reduces the interaction between SlNAP1 and SlGID1, exogenous GA intensifies it (Additional file 1: Fig. S8). These results indicate that the interaction between SlNAP1 and GID1 plays a crucial role in the process of SlNAP1 promoting tomato FR by regulating the transcription level of targeted genes and thereby inhibiting GA synthesis. However, the roles played by ABA and other phytohormone pathways, as well as the connections between GID1 protein and SlNAP1 in the ripening process, continue to pose questions in need of clarification.

## Conclusions

On the basis of our findings we have deduced that SlNAP1, a NAC TF in tomato, is involved in FR. Our research demonstrated that SlNAP1 has the capability to directly bind to and stimulate the promoter of both *SlGA2ox1* and *SlGA2ox5*. In addition, we have effectively validated the interaction between SlNAP1 and SlGID1 during FR of tomato. Collectively, this study on tomato NAC TF SlNAP1 reveals that SlNAP1 promotes FR as an activator by directly suppressing GA biosynthesis genes and directly activating GA degradation genes, and the crosstalk between SlNAP1 and SlGID4. By modifying the regulatory elements that initiate and regulate senescence, manipulating FR becomes a potent strategy for enhancing agricultural productivity, particularly in the cultivation of fleshy fruits.

### Supplementary Information

**Additional file 1: Figure S1**. SlNAP1 protein belongs to the NAC transcription factor (TF) family. **Figure S2**. A phylogenetic tree showing the evolutionary relationships of the subgenus families, including the known genes involved in tomato fruit ripening. **Figure S3**. Fruit phenotype and *SlNAP1* gene expression of TRV and TRV-*SlNAP1*. **Figure S4.** Ethylene content and the expression levels of ethylene synthesis-related genes *ACC oxidase 1 *(*SlACO1*), *ACC oxidase 1* (*SlACO3*), *ACC synthase 2* (*SlACS2*), and *ACC synthase 4* (*SlACS4*) in wild-type (WT), *slnap1-5*, and *slnap1-16* fruits at mature green (MG), breaker (Br), 4 days after the breaker (Br + 4), and 7 days after the breaker (Br + 7) stages. ACC,1-aminocyclopropyl 1-carboxylic acid. **Figure S5**. Chlorophyll (Chl) and carotenoid (Car) content in wild-type (WT), *slnap1-5*, and *slnap1-16* fruits. **Figure S6**. The expression levels of chlorophyll (Chl) degradation-related genes *non-yellow coloring1* (*SlNYC1*), *stay green 1* (*SlSGR1*), *pheophide a oxygenase* (*SlPAO*), *pheophytinase* (*SlPPH*) and *red chlorophyll catabolite reductase* (*SlRCCR*) in wild-type (WT), *slnap1-5*, and *slnap1-16* fruits at mature green (MG) stage. **Figure S7**. The expression levels of carotenoid (Car) synthesis-related genes *phytoene synthase 1* (*SlPSY1*; A), *phytoene synthase 2* (*SlPSY2*; B), *phytoene synthase 3* (*SlPSY3*; C), *copalyl diphosphate synthases* (*SlCPPS*; D), *isoprenyl diphosphate synthases* (*SlIDS*; E), *lycopene ß-cyclase 1* (*SlLCYB1*; F), *lycopene ß-cyclase 2 *(*SlLCYB2*; G), *lycopene δ-cyclase* (*SlLCYE*; H), *β-carotene hydroxylase 1* (*SlCYHB1*; I), *β-carotene hydroxylase 2* (*SlCYHB2*; J), *violaxanthin deepoxidase* (*SlVED*; K), *zeaxanthin epoxidase* (*SlZFP*; L) in wild-type (WT), *slnap1-5*, and *slnap1-16* fruits at breaker (Br), 4 days after the breaker (Br + 4), and 7 days after the breaker (Br + 7) stages. **Figure S8**. The effect of gibberellin (GA) on the interaction between SlNAP1 and SlGID1. **Table S1**. Primers used for vector construction. **Table S2**. Primers used for quantitative real-time PCR (qRT-PCR). **Table S3**. Primers used for target site mutation analysis.

## Data Availability

Data will be available upon request.

## Abbreviations

LUC: Luciferase
ETH: Ethylene
ABA: Abscisic acid
TF: Transcription factor
Nor: Nonripening
CDS: Coding sequence
Car: Carotenoid
BR: Brassinolide
MT: Melatonin
RIN: Ripening inhibitor
CNR: Colorless nonripening
FUL1/2: FRUITFULL1/2
FYFL: Forever young flower
Chl: Chlorophyll
GID1: Gibberellin-insensitive dwarf1
WT: Wild-type
TAIR: The *Arabidopsis* Information Resource
SGN: Sol Genomics Network
NJ: Neighbor-joining
MES: 2-(4-Morpholino) ethanesulfonic acid
AS: Acetosyringone
PAC: Paclobutrazol
VIGS: Virus induced gene silencing
HPLC: High-performance liquid chromatography
AbA: Aureobasidin A
SD: Standard deviation
BD: DNA-binding domain
AD: DNA-activating domain
RNAi: RNA interference
PSY1: Phytoene synthase 1
YAB1: YABBY1 gene
SGD2: Small Grain and Dwarf 2
PRO: PROCERA
