# Supplementary material for: A tomato NAC transcription factor, SlNAP1, directly regulates gibberellin-dependent fruit ripening
Source: Cell Mol Biol Lett. 2024 Apr 23;29:57. doi: 10.1186/s11658-024-00577-7 (PMC11036752; doi:10.1186/s11658-024-00577-7)
Supplement: Supplementary file 1 — Additional file 1: Figure S1. SlNAP1 protein belongs to the NAC transcription factor (TF) family. Figure S2. A phylogenetic tree showing the evolutionary relationships of the subgenus families, including the known genes involved in tomato fruit ripening. Figure S3. Fruit phenotype and SlNAP1 gene expression of TRV and TRV-SlNAP1. Figure S4. Ethylene content and the expression levels of ethylene synthesis-related genes ACC oxidase 1 (SlACO1), ACC oxidase 1 (SlACO3), ACC synthase 2 (SlACS2), and ACC synthase 4 (SlACS4) in wild-type (WT), slnap1-5, and slnap1-16 fruits at mature green (MG), breaker (Br), 4 days after the breaker (Br + 4), and 7 days after the breaker (Br + 7) stages. ACC,1-aminocyclopropyl 1-carboxylic acid. Figure S5. Chlorophyll (Chl) and carotenoid (Car) content in wild-type (WT), slnap1-5, and slnap1-16 fruits. Figure S6. The expression levels of chlorophyll (Chl) degradation-related genes non-yellow coloring1 (SlNYC1), stay green 1 (SlSGR1), pheophide a oxygenase (SlPAO), pheophytinase (SlPPH) and red chlorophyll catabolite reductase (SlRCCR) in wild-type (WT), slnap1-5, and slnap1-16 fruits at mature green (MG) stage. Figure S7. The expression levels of carotenoid (Car) synthesis-related genes phytoene synthase 1 (SlPSY1; A), phytoene synthase 2 (SlPSY2; B), phytoene synthase 3 (SlPSY3; C), copalyl diphosphate synthases (SlCPPS; D), isoprenyl diphosphate synthases (SlIDS; E), lycopene ß-cyclase 1 (SlLCYB1; F), lycopene ß-cyclase 2 (SlLCYB2; G), lycopene δ-cyclase (SlLCYE; H), β-carotene hydroxylase 1 (SlCYHB1; I), β-carotene hydroxylase 2 (SlCYHB2; J), violaxanthin deepoxidase (SlVED; K), zeaxanthin epoxidase (SlZFP; L) in wild-type (WT), slnap1-5, and slnap1-16 fruits at breaker (Br), 4 days after the breaker (Br + 4), and 7 days after the breaker (Br + 7) stages. Figure S8. The effect of gibberellin (GA) on the interaction between SlNAP1 and SlGID1. Table S1. Primers used for vector construction. Table S2. Primers used for quantitative real-tim [file 11658_2024_577_MOESM1_ESM.zip › Supplementary/Table S2.docx]

**Table S2** Primers used for qRT-PCR.

| Experiment | Primer name | Sequence from 5’ to 3’ |
| --- | --- | --- |
| qRT-PCR | *SlACO1* F | CCACCATGTCCTAAGCCCGATTTG |
|  | *SlACO1* R | TTGCTCGTCTTTGAGGAGTTGAAGG |
|  | *SlACO3* F | CCACCATGTCCTAAGCCCGATTTG |
|  | *SlACO3* R | TTGCTCGTCTTTGAGGAGTTGAAGG |
|  | *SlACS2* F | GCTTAACGTCTCGCCTGGATCTTC |
|  | *SlACS2* R | CTCAACACCTACGAACCTCCGAATC |
|  | *SlACS4* F | TTGGATGGATTTGCGGTCATTGTTG |
|  | *SlACS4* R | CAGGGGAGACGTTGAGCTTAACT |
|  | *SlNYC1* F  *SlNYC1* R | CCTAACCGACCTACTTCTGAGTGG  AGCAACTGTCTCTGGATGTTCGC |
|  | *SlSGR1* F  *SlSGR1* R | CCAGTGAGTGTTATGCCTTGG  TCAACTTTGCTGCTCTTGCAAG |
|  | *SlPPH* F | CCCATGATGAAGTCCCAGAGATTG |
|  | *SlPPH* R | ATCGGATTCAACGTTCCTGATCC |
|  | *SlPAO* F | CATGGAGAACGTCTCTGATCCTTC |
|  | *SlPAO* R | TGTCTCGCCTTCCAGTAACCTTG |
|  | *SlRCCR* F | ATTCGAGCCCTTGTCTCTCCATC |
|  | *SlRCCR* R | TCAATGCGAATGGCCTGGGAAG |
|  | *SlPSY1* F | GGAATGCGTATGGACTTGAGAA |
|  | *SlPSY1* R | GGATAGACCTGCCTGTGCTAA |
|  | *SlPSY2* F | GAATGCGTATGGACTTGTGGAA |
|  | *SlPSY2* R | CCTGCCTGTGCTAATTCATCTT |
|  | *SlPSY3* F | GACATCAAGCCGTTCAAGGACA |
|  | *SlPSY3* R | GCCAACTGTTCCAGCCACAC |
|  | *SlCPPS* F | CCATGCCACGACCAGAAGATTG |
|  | *SlCPPS* R | GCCTCCAGCAGTATCGGTTTGT |
|  | *SlIDS* F | ACATGAGTGCTCGGTGTATGCT |
|  | *SlIDS* R | TGCCTCGAACGCTCCAAGTC |
|  | *SlLCYB1* F | GAGAGTCGTTGGATCGGTGGTA |
|  | *SlLCYB1* R | ATGGCATTGGCAACAACAGGAG |
|  | *SlLCYB2* F | GGATTGCCTTGATGCGACAT |
|  | *SlLCYB2* R | ATGCCATAAGCGACTTGATACC |
|  | *SlLCYE* F | TGGTCCTGCTGGTCTTGCTCTT |
|  | *SlLCYE* R | ACACCTGCCTCCACACACCTT |
|  | *SlCYHB1* F | GCTGCTGCTCATTCGCTTCATC |
|  | *SlCYHB1* R | TTCCAACTCTTCCGTCCCTCCT |
|  | *SlCYHB2* F | ACCATTCGCCTCCGTCATAACC |
|  | *SlCYHB2* R | CCAATTTCTCCGCCAGCCAACT |
|  | *SlVED* F | GCTGATGCCGTTGATGCTCTC |
|  | *SlVED* R | TCAGGCACAGGAAAGTCACCAA |
|  | *SlZEP* F | CCAGCAGTGGAACGTGGACTT |
|  | *SlZEP* R | GCCATCAGCACCAACCAGAAGA |
|  | *SlGA2ox1* F | CGTATCACCTCACCCGAATACTGC |
|  | *SlGA2ox1* R | GCCCTATGCCTCACACTCTTAAACC |
|  | *SlGA2ox5* F | CCTGACTCCAAGAACCTCATCGTTG |
|  | *SlGA2ox5* R | GGGGAGAGGAGAAGAATTTGATGGC |
